# Cesarean section history affects the outcomes of frozen embryo transfer in IVF/ICSI: a retrospective study

**DOI:** 10.3389/fsurg.2025.1671347

**Published:** 2025-10-09

**Authors:** Xiaohui Wang, Yan Zhou

**Affiliations:** 1 Department of Reproductive Medicine, Women's Hospital School of Medicine Zhejiang University, Hangzhou, Zhejiang, China; 2 Department of Obstetrics and Gynecology, Henan Provincial People’s Hospital, Zhengzhou University People’s Hospital, Zhengzhou, Henan, China

**Keywords:** cesarean section, scar defect, frozen embryo transfer, *in vitro* fertilization, pregnancy outcome

## Abstract

**Objective:**

Cesarean section (CS) rates have risen globally, with cesarean scar defect (CSD) being a common complication. Few studies have investigated the impact of CSD on *in vitro* fertilization/intracytoplasmic sperm injection frozen embryo transfer (IVF/ICSI-FET) outcomes. This study assessed how prior CS history (with or without CSD) affects IVF/ICSI-FET outcomes compared with prior vaginal delivery (VD).

**Methods:**

We retrospectively analyzed 985 IVF/ICSI-FET patients: 597 patients with prior VD (VD group) and 388 with prior CS (CS group). The CS group was subdivided into those without CSD (NCSD, *n* = 283) and those with CSD (CSD, *n* = 105). Binary logistic regression was used to assess the associations between delivery history and pregnancy outcomes.

**Results:**

No significant differences in early abortion, premature birth, perinatal complications or birth weight were detected between the VD and CS groups. However, compared with the VD group, the CS group had significantly lower biochemical pregnancy, implantation, live birth, and clinical pregnancy rates. Among women with prior CS, the CSD group had significantly higher rates of premature birth and perinatal complications than the NCSD group did. The size of the CSD did not affect the live birth rate or clinical pregnancy rate.

**Conclusions:**

Prior CS negatively affects IVF/ICSI-FET pregnancy outcomes. The presence of CSD further increases premature birth and perinatal complication rates.

## Introduction

Cesarean section (CS) rates have risen globally. In China, the CS rate is increasing and remains high. From 2008 to 2018, the CS rate increased from 28.8% to 36.7% (1), which is higher than the reasonable range of 10%–15% stipulated by the World Health Organization (WHO) (2). The common complications of CS include infection, postpartum hemorrhage, and complications of subsequent pregnancies, such as scar pregnancy, placental implantation and uterine rupture (3). Various studies have shown that a previous CS might reduce the subsequent pregnancy rate and prolong pregnancy intervals (4–6).

Cesarean section defects (CSDs) are among the common complications of CS. CSDs were first proposed by Morris in 1995 (7). Any myometrial defect in the lower portion of the uterus after uterine surgery can be considered a CSD. Most CSDs are asymptomatic. Some patients may develop complications such as abnormal uterine bleeding, scar pregnancy, late pregnancy uterine rupture, and postpartum hemorrhage. CSDs are observed in 24%–70% of women after CS when assessed by transvaginal sonography (TVS) (8, 9). The accumulation of mucus and blood at the scar affects embryo transfer, implantation and sperm upstream. The presence of a CSD may also increase the difficulty of embryo transfer and affect the success rate of IVF (4, 10–12).

Currently, the number of secondary infertility patients with a history of CS who choose *in vitro* fertilization/intracytoplasmic sperm injection-embryo transfer (IVF/ICSI-ET) as an alternative strategy for their progeny has markedly increased. However, the influence of CS on pregnancy outcomes after assisted reproductive technology is still controversial (10, 13–16). Previous studies have reported conflicting results on the impact of CS and CSD on embryo transfer outcomes. Some studies showed that CS reduced live birth rate and clinical pregnancy rate and increased miscarriage risk (8–12), while others found no significant association, especially with single embryo transfer (13). Similarly, several studies, including a systematic review, suggested that CSD, particularly large defects, impairs pregnancy outcomes in ART cycle (17–19), whereas Wu et al. reported no effect of CSD size in fresh embryo transfer cycles (20). These inconsistencies highlight the need for further clarification in standardized FET cycles. The purpose of this study was to investigate the influence of a previous CS and CSD on the pregnancy outcome of patients undergoing IVF/ICSI frozen-thawed embryo transfer (FET) cycles.

## Methods

### Patient population

This retrospective study included patients with only one previous CS who underwent IVF/ICSI FET at our reproductive center of Women's Hospital School of Medicine, Zhejiang University, from January 2015 to December 2019. Ethical approval was granted by the Ethics Committee of Women's Hospital, Zhejiang University School of Medicine. The inclusion criteria were as follows: (1) underwent FET cycles; (2) received either a gonadotropin-releasing hormone (GnRH) agonist (GnRH-a) or a GnRH antagonist (GnRH-ant) ovarian stimulation protocol; (3) underwent an IVF/ICSI cycle; (4) were aged between 20 and 40 years; and (5) were treated with a hormone replacement therapy (HRT) cycle. The exclusion criteria were as follows: (1) a history of unilateral oophorectomy, myomectomy or resection of an endometrioma; (2) congenital or acquired uterine malformations, endometrioma, adenomyosis, or intrauterine adhesion; (3) chromosomal abnormalities; (4) a history of recurrent spontaneous abortion (defined as two or more previous spontaneous pregnancy losses); (5) BMI > 28 kg/m^2^; or (6) missing records in the electronic database. In accordance with the above criteria, a total of 985 patients were included in the study.

Transvaginal sonography (TVS) is used to determine whether a patient has a previous CSD. In accordance with some criteria, CSD is defined as an anechoic defect of at least 2.0 mm in depth (21). TVS can be used to evaluate the size of the CSD by determining its depth, width, length and residual myometrial thickness (RMT) in the median sagittal plane and cross-section. A large CSD is defined as when the RMT is <50% of the adjacent myometrium or ≤2.2 mm on TVS (22).

### IVF/ICSI protocol

All patients underwent a GnRH-a protocol or a GnRH-ant protocol under controlled ovarian stimulation. Transvaginal oocyte retrieval was performed 34–36 h after the HCG injection. In our hospital, oocyte retrieval and *in vitro* embryo culture are carried out by embryologists in turn according to a fixed schedule. The oocytes are fertilized with sperm, after which IVF or ICSI is performed. The embryos were cultured *in vitro* for 2 or 3 days, after which high-quality embryos were cryopreserved.

In the FET cycle, all patients received hormone replacement therapy (HRT) to prepare the endometrium. From the 2nd day to the 3rd day of menstruation, estradiol valerate was given at a dosage of 2–3 mg/2 times a day, and 17β-estradiol tablets were added according to the thickness of the endometrium. When the standard endometrial thickness was reached, dydrogesterone and progesterone soft capsules were added to transform the endometrium. Transplantation was performed on the 3rd or 5th day according to the embryo stage.

Single-embryo transfer is recommended for patients with a history of CS, but some patients insist on double-embryo transfer. Therefore, we divided the transferred embryos into 6 groups: (1) a blastocyst; (2) double high-quality cleavage embryos; (3) single good cleavage embryo and single poor cleavage embryo; (4) double low-quality cleavage embryos; (5) single high-quality cleavage embryo; (6) single low-quality cleavage embryo.

### Observation indicators and main outcome measures

Basic clinical data included age, body mass index (BMI), infertility duration, infertility cause, endometrial thickness and number of follicles with a diameter ≥14 mm on the trigger day, bFSH/LH, number of embryos transferred, time since the previous CS and quality of the transplanted embryos. The primary outcome measure was clinical pregnancy. Prespecified secondary outcomes included biochemical pregnancy, live birth, implantation rate, early abortion, premature birth and neonatal weight.

We defined clinical pregnancy as the observation of a gestational sac with or without a fetal heartbeat by TVS. Biochemical pregnancy was identified as an increased serum hCG concentration (≥5 IU/L) 14 days following FET. Live birth was defined as the delivery of a viable infant with signs of life after 24 completed weeks of gestation. The implantation rate was defined as the ratio of the number of amniotic sacs per patient to the number of embryos transferred per patient. The early abortion was defined as spontaneous abortion within 12 weeks of pregnancy (excluding biochemical pregnancy). In cases of twin pregnancies where only one sac was lost but the other continued to develop, the pregnancy was not classified as a miscarriage. Premature birth refers to newborns born before 37 complete weeks.

### Statistical analysis

Statistical analysis was performed using SPSS 26.0 software. The normality of variables should be evaluated to determine whether parametric or nonparametric statistical methods should be used. Continuous variables are presented as the means and standard deviations. Student's *t*-test and the Mann‒Whitney *U* test were used to compare the central tendencies of the parametric and nonparametric variables, respectively. Categorical variables are presented as numbers with rates, and the *χ*^2^ test and Fisher's exact test were used for comparisons. One-way ANOVA and the SNK-q test were used for the intergroup comparisons. Binary logistic regression analyses were performed to assess the association between the previous mode of delivery and FET pregnancy outcomes. Crude and adjusted odds ratios (ORs) with 95% confidence intervals (95% CI) were calculated before and after adjusting for confounding variables. Confounding variables with a *p* value of 0.1 and known clinical relevance were retained in the adjusted model, including age, BMI, the number of embryos transferred, the infertility cause, endometrial thickness, and quality of transplanted embryos. All significance analyses were two-sided and tested at the 5% level. *P* < 0.05 was considered to indicate statistical significance.

## Results

### Baseline characteristics of the study population

**Figure 1:**
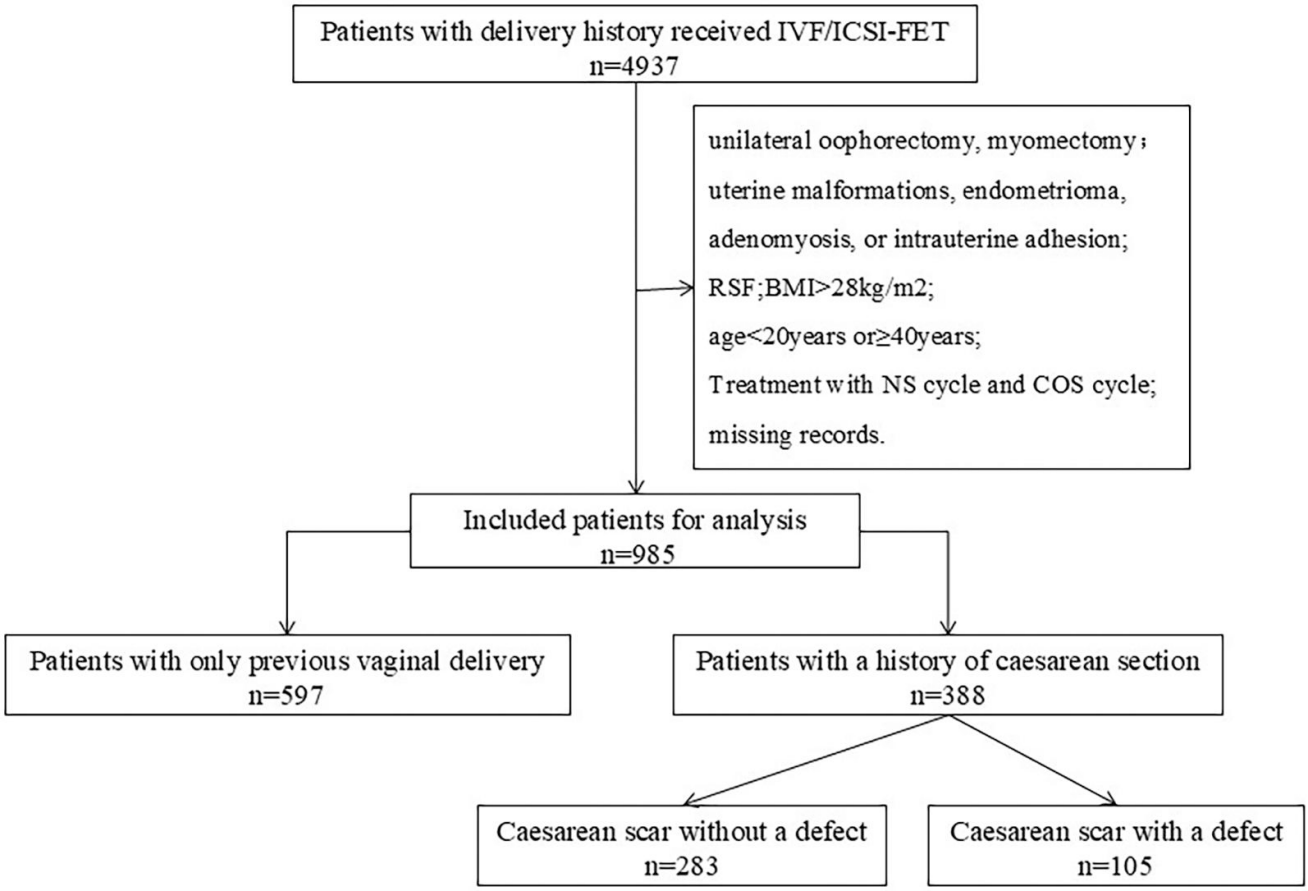
Study flowchart. Flowchart of patient selection and grouping. Patients who underwent IVF/ICSI-FET cycles from 2015 to 2019 were retrospectively screened. The exclusion criteria included repeated cycles, uterine anomalies, and missing data. Eligible patients were divided into a vaginal delivery (VD) group and a cesarean section (CS) group, with the CS group further subdivided into cesarean scar without a defect (NCSD) and cesarean scar defect (CSD) subgroups.

A total of 985 patients were included in this study (Figure 1). Depending on the mode of delivery, the patients were divided into two groups: the vaginal delivery (VD) group (*n* = 597) and the CS group (*n* = 388). We further divided the CS group into two subgroups according to the presence of a cesarean scar: a cesarean scar without a defect (NCSD) group (*n* = 283) and a cesarean scar with a defect (CSD) group (*n* = 105). The baseline characteristics of the women are shown in Table 1.

**Table 1:** Baseline characteristics of different groups.

Parameter	VD (*n* = 597)	CS (*n* = 388)	*P*	NCSD (*n* = 283)	CSD (*n* = 105)	*P*
Age (years)	32.71 ± 3.76	33.12 ± 4.01	0.056	33.12 ± 4.01	33.08 ± 4.01	0.317
endometrial thickness (mm)	9.99 ± 1.71	9.85 ± 1.64	0.279	9.85 ± 1.64	9.87 ± 1.64	0.418
Numbers of follicles with a diameter ≥ 14 mm on the trigger day	10 (8, 13)	10 (7, 12.25)	0.071	10 (7, 12)	10 (7, 13)	0.110
Infertility duration (years)	3.737 ± 0.114	3.638 ± 2.635	0.690	3.445 ± 0.157	4.160 ± 0.251	0.002*
bFSH/LH	1.51 ± 0.80	1.47 ± 0.73	0.408	1.35 ± 0.53	1.59 ± 0.75	0.011*
BMI (kg/m^2^)	22.30 ± 2.46	22.42 ± 2.31	0.345	22.56 ± 2.24	22.04 ± 2.45	0.058
underweight < 18.5 kg/m^2^	30 (5.0%)	13 (3.3%)		7 (2.4%)	6 (5.7%)	
normal weight 18.5–23.9 kg/m^2^	412 (69.0%)	282 (72.6%)		204 (72.0%)	78 (74.2%)	
overweight 24–27.9 kg/m^2^	155 (25.9%)	93 (23.9%)		72 (25.4%)	21 (20%)	
Infertility cause			0.015*			0.006*
Tubal factor	468 (78.4%)	285 (73.5%)		208 (73.5%)	77 (73.3%)	
Male factor	61 (10.2%)	48 (12.4%)		38 (13.4%)	10 (9.5%)	
Endometriosis	9 (1.5%)	12 (3.1%)		7 (2.5%)	5 (4.8%)	
Unexplained	28 (4.7%)	14 (3.6%)		11 (3.9%)	3 (2.9%)	
DOR	3 (0.5%)*	11 (2.8%)*		3 (1.1%)*	8 (7.6%)*	
Other factors	28 (4.7%)	18 (4.6%)		16 (5.7%)	2 (1.9%)	
Numbers of embryos transferred (*n*)			0.612			0.976
1	168 (28.1%)	115 (29.6%)		84 (73%)	199 (72.9%)	
2	429 (71.9%)	273 (70.4%)		31 (27%)	74 (27.1%)	
Quality of transplanted embryos (*n*)			0.929			0.658
Class 1	51 (8.5%）	31 (7.90%)		25 (8.8%）	6 (5.7%）	
Class 2	242 (40.5%)	16 (41.49%)		113 (39.9%)	48 (45.7%)	
Class 3	84 (14.1%）	47 (12.11%)		36 (12.7%)	11 (10.5%)	
Class 4	93 (15.6%)	62 (16.00%)		47 (16.6%)	15 (14.3%)	
Class 5	75 (12.6%）	49 (12.63%)		37 (13.1%)	12 (11.4%)	
Class 6	52 (8.7%）	38 (9.79%)		25 (8.8%)	13 (12.4%)	
Time since previous CS(years)				8.33 ± 3.771	8.75 ± 3.275	0.088

* *P* < 0.05.

Class1, a blastocyst; Class2, double high-quality cleavage embryos; Class3, single good cleavage embryo and single poor cleavage embryo; Class4, double low-quality cleavage embryos; Class5, single high-quality cleavage embryo; Class6, single low-quality cleavage embryo.

VD, caginal delivery; CS, caesarean section; NCSD, caesarean scar without a defect; CSD, caesarean scar defect; bFSH/LH, basal Follicle-stimulating hormone/luteinizing hormone ratio; BMI, body mass index; DOR, diminished ovarian reserve.

For the VD group and the CSD group, there were no significant differences in age, BMI, bFSH/LH, endometrial thickness, number of follicles with a diameter ≥14 mm on the trigger day, number of embryos transferred, infertility duration, or quality of transplanted embryos between the VD group and the CS group (*P* > 0.05). However, the cause of infertility was significantly different between the two groups (*P* = 0.015), and diminished ovarian reserve (DOR) was significantly different between the two groups (0.5% vs. 2.8%).

For the NCSD group and the CSD group, we found no significant difference in age, BMI, endometrial thickness, number of follicles with a diameter ≥14 mm on the trigger day, number of embryos transferred, time since the previous CS, or quality of the transplanted embryos (*P* > 0.05). The infertility duration, infertility cause and bFSH/LH ratio significantly differed between the two groups (*P* < 0.05). Among the infertility causes, DOR was significantly different between the NCSD group and the CSD group (1.1% vs. 7.6%). Single-factor regression analysis revealed that these factors had no effect on pregnancy outcomes.

### Pregnancy outcomes after embryo transfer

#### Cesarean section vs. vaginal delivery

The pregnancy outcomes of the VD group and the CS group are shown in Table 2. There was no significant difference in the early abortion rate, premature birth rate, or birth weight or low birth weight rate (*P* > 0.05). The difference in the implantation rate between the two groups was significant (*P* *=* 0.02). The biochemical pregnancy rate in the CS group was significantly lower than that in the VD group [42.3% vs. 52.4%, crude OR, 0.664 [0.513, 0.86], adjusted OR, 0.690 [0.529, 0.900], *P* = 0.006]. The live birth rate in the CS group was significantly lower than that in the VD group [27.32% vs. 36.85%, crude OR, 0.644 [0.488, 0.851], adjusted OR, 0.662 [0.497, 0.882], *P* = 0.005]. The clinical pregnancy rate was also significantly different between the CS group and the VD group [36.6% vs. 47.4%, crude OR, 0.648 [0.499, 0.841], adjusted OR, 0.665 [0.508, 0.871], *P* = 0.003]. We also analyzed the differences in perinatal complications, including gestational diabetes mellitus (GDM), hypertensive disorders of pregnancy (HDP) and placenta previa, between the two groups. The perinatal complication rate in the CS group was higher than that in the VD group (11.32% vs. 7.27%), but the difference was not significant (*P* = 0.201).

**Table 2:** Pregnancy outcomes in women with or without CS.

Parameter	VD (*n* = 597)	CS (*n* = 388)	OR	*P*	Adjusted OR^a^	*P*
Biochemical pregnancy	52.4% (313/597)	42.3% (164/388)	0.664 (0.513, 0.860)	0.002*	0.690 (0.529, 0.900)	0.006*
Implantation rate	0.334 ± 0.016	0.251 ± 0.018	0.427 (0.174, 0.680)	0.01*	0.401 (0.144, 0.659)	0.02*
Live birth	36.85% (220/597)	27.32% (106/388)	0.644 (0.488, 0.851)	0.002*	0.662 (0.497, 0.882)	0.005*
Clinical pregnancy	47.40% (283/597)	36.60 (142/388)	0.648 (0.499, 0.841)	0.01*	0.665 (0.508, 0.871)	0.003*
Early abortion rate	17.31% (49/283)	21.83% (31/142)	1.322 (0.799, 2.186)	0.277	1.339 (0.795, 2.255)	0.273
Premature birth rate	28.64% (63/220)	16.04% (17/106)	0.723 (0.392, 1.333)	0.298	0.826 (0.435, 1.569)	0.560
Birth weight (kg)	3.15 (2.69,3.50)	3.1 (2.84,3.45)	–	–	–	0.537
Low birth weight rate	18.94 (50/264)	12.71% (15/118)	0.605 (0.325, 1.127)	0.114	1.474 (0.749, 2.898)	0.261
Perinatal complications rate	7.27% (16/220)	11.32% (12/106)	1.628 (0.741, 3.577)	0.225	1.670 (0.761, 3.662)	0.201

* *P* < 0.05.

a Adjusted for confounding age, BMI, the number of embryos transferred, the infertility cause, endometrial thickness, and quality of transplanted embryos.

VD, vaginal delivery; CS, caesarean section.

#### CSD vs. NCSD among women with prior CS

We further divided patients who had previous CS into groups with or without cesarean scar defects. Table 3 presents the pregnancy outcomes between the NCSD group and the CSD group. There were no significant differences in the implantation rate, biochemical pregnancy rate, live birth rate, early abortion rate, birth weight or low birth weight between the two groups (*P* > 0.05). The biochemical pregnancy rate (25.6% vs. 43.1%) and low birth weight rate (17.9% vs. 10.1%) in the CSD group were higher than those in the NCSD group, but the difference was not significant (*P* *>* 0.05). The premature birth rate in the CSD group was higher than that in the NCSD group [30.8% vs. 11.3%, crude OR, 3.505 [1.186, 10.363], adjusted OR, 3.504 [1.079, 11.377], *P* = 0.037]. The perinatal complication rate was also significantly different between the NCSD group and the CSD group (30.8% vs. 13.8%, *P* = 0.049).

**Table 3:** Subgroup analysis of cesarean section cases according to the presence of a scar defect.

Parameter	NCSD (*n* = 283)	CSD (*n* = 105)	OR	*P*	Adjusted OR^a^	*P*
Biochemical pregnancy	43.1% (122/283)	25.6% (42/105)	0.880 (0.558, 1.388)	0.582	1.124 (0.692, 1.827)	0.637
Implantation rate	0.251 ± 0.360	0.252 ± 0.360	0.782 (0.491, 1.246)	0.301	0.848 (0.371, 1.525)	0.502
Live birth	28.3% (80/283)	24.8% (26/105)	0.686 (0.429, 1.097)	0.116	1.206 (0.703, 2.069)	0.496
Clinical pregnancy	38.5% (109/283)	32.4% (34/105)	0.764 (0.476, 1.228)	0.266	1.315 (0.798, 2.167)	0.283
Early abortion rate	22.9% (25/109)	17.6% (6/34)	0.720 (0.268, 1.934)	0.515	2.038 (0.699, 5.945)	0.192
Premature birth rate	11.3% (9/80)	30.8% (8/26)	3.505 (1.186, 10.363)	0.023*	3.504 (1.079, 11.377)	0.037*
Birth weight (kg)	3.23 (2.835,3.6)	3.05 (2.63,3.29)				0.077
Low birth weight rate	10.1% (9/89)	17.9% (5/28)	1.932 (0.589,6.336)	0.277	1.435 (0.382, 5.393)	0.593
Perinatal complications rate (%)	13.8% (11/80)	30.8% (8/26)	–	–	–	0.049*
GDM	8.8% (7/80)	26.9% (7/26)	–	–	–	–
HDP	5% (4/80)	7.7% (2/26)	–	–	–	–
Placenta previa	0	3.8% (1/26)	–	–	–	–

* *P* < 0.05.

a Adjusted for confounding age, BMI, the number of embryos transferred, the infertility cause, endometrial thickness, and quality of transplanted embryos.

NCSD, caesarean scar without a defect; CSD, caesarean scar defect; GDM, gestational diabetes mellitus; HDP, hypertensive disorders of pregnancy; OR, odds ratio.

#### Pregnancy outcomes according to CSD size

Furthermore, we studied the influence of diverticulum size on pregnancy outcomes. A large CSD is defined as an RMT ≤2.2 mm on the TVS. We compared the pregnancy outcomes of patients without CSDs and patients with large or small CSDs. There were no significant differences in the live birth rate or clinical pregnancy rate among the NCSD patients, the patients with large CSDs, or the patients with small CSDs (*P* > 0.05) (Table 4).

**Table 4:** Pregnancy outcomes in women with large CSD (RMT ≦ 2.2 mm).

Group	% (*n*/*N*)	OR	*P*	Adjusted OR^a^	*P*
Live birth					
NCSD; *N* = 283	28.3% (80/283)	1			
CSD (>2.2 mm); *N* = 83	22.8% (19/83)	1.559 (0.930–2.612)	0.093	0.578 (0.216–1.548)	0.276
CSD (≦2.2 mm); *N* = 22	31.8% (7/22)	0.636 (0.226–1.787)	0.391	0.398 (0.132–1.197)	0.101
Clinical pregnancy					
NCSD; *N* = 283	38.5% (109/283)	1			
CSD (>2.2 mm); *N* = 83	32.5% (27/83)	1.610 (0.637–4.072)	0.314	1.114 (0.418–2.970)	0.828
CSD (≦2.2 mm); *N* = 22	31.8% (7/22)	1.033 (0.377–2.831)	0.949	0.827 (0.283–2.416)	0.729

a Adjusted for confounding age, BMI, the number of embryos transferred, the infertility cause, endometrial thickness, and quality of transplanted embryos.

NCSD, caesarean scar without a defect; CSD, caesarean scar defect; RMT, residual myometrial thickness; OR, odds ratio.

## Discussion

In this retrospective study, we investigated the relationship between the previous delivery mode and the subsequent pregnancy outcome of patients who received IVF/ICSI-FET. The implantation rate, biochemical birth rate, live birth rate and clinical pregnancy rate of the CS group were significantly lower than those of the VD group. Among patients with a previous CS, the premature delivery rate and perinatal complication rate were significantly higher in the CSD group than in the CSD group. And we found no difference of diverticulum size on pregnancy outcomes. Our findings align with reports that prior CS lowers pregnancy and live birth rates (8, 10–12, 17), although other studies found no effect (13). For CSD, most evidence suggests adverse effects, especially with large defects (17–19), but some studies found no association (20). Differences in study design, embryo stage, and CSD definitions may explain these discrepancies. By focusing on HRT-FET cycles, our study adds evidence that CS history impairs outcomes, while CSD mainly increases risks in later pregnancy rather than affecting implantation.

We screened all FET patients who met the inclusion criteria and received IVF/ICSI from 2015 to 2019. All patients were treated with HRT for endometrial preparation. In our study, some significant differences were detected in the baseline characteristics of some patients, such as infertility duration, bFSH/LH ratio and causes of infertility. However, we found that these parameters had no effect on pregnancy outcomes after single-factor regression analysis, which made the results of this study more credible.

After adjusting for confounding factors, our study revealed that the implantation rate, biochemical pregnancy rate, clinical pregnancy rate and live birth rate of the CS group were significantly lower than those of the VD group. Several studies reported that a prior CS was associated with a decreased likelihood of live birth in subsequent IVF, which was consistent with the findings of our study (9, 13, 20). However, their study focused exclusively on fresh embryo transfer cycles and revealed no significant difference between women with and without CSD, suggesting that the impact of uterine scarring may vary depending on the transfer type and endometrial preparation. In contrast, our study specifically analyzed frozen embryo FET cycles with standardized HRT protocols, which may better reveal the adverse effects of CSD by minimizing the confounding effects of ovarian stimulation on endometrial receptivity. These results suggest that the presence of a CS scar may change the contractility of the myometrium and disrupt the normal contractile wave of the endometrium, thus affecting embryo implantation and increasing the difficulty of embryo transfer. Moreover, local inflammation of the cesarean scar affects the pregnancy outcome of IVF/ICSI-FET (10, 16, 23, 24). All this pathological evidence could explain our results regarding the decreased live birth rate in IVF/ICSI after a previous CS. Our study revealed no difference in birth weight between the VD group and the CS group, which is consistent with a recent retrospective study (25). However, some studies have reported inconsistent conclusions. They revealed that a previous CS did not affect pregnancy outcomes among IVF cycles (24, 26). The sample size of this study was small, and there were significant differences from the baseline, which might have affected the outcomes. A prospective cohort study also showed that CS did not affect pregnancy outcomes, such as live births (39% vs. 32%) and clinical pregnancy (49% vs. 41%). However, they stopped the research before they recruited the planned number of patients, which limited its ability to detect relevant differences (16).

We also reported that the perinatal complication rate was higher in the CS group than in the VD group. We further analyzed the perinatal complication rate of patients with a previous CS history and reported that the rate in the CSD group was significantly greater than that in the NCSD group. This conclusion was consistent with that of a previous study (10). Thus, for patients with a prior CS, especially those with CSD in the second and third trimesters of pregnancy, we should strictly strengthen pregnancy monitoring and risk control to address pregnancy complications as early as possible and maintain pregnancy safety.

The effect of CSD on IVF/ICSI-FET outcomes remains controversial. In our study, we found no difference in implantation or pregnancy establishment rates, but obstetric outcomes after pregnancy were worse in the CSD group. Our research revealed that the premature birth rate and perinatal complications were higher in the CSD group than in the NCSD group. To avoid obstetric complications during the late stage of pregnancy, some patients with CSDs had to terminate pregnancy before a full-term pregnancy. This factor might have led to a higher premature birth rate in the CSD group than in the NCSD group to a certain extent. However, the sample size was small, and we were not aware of the reasons for previous CS and recovery after the operation, which might have reduced the statistical power and increased the risk of type II error, thus affecting the research results.

On the other hand, we found no significant difference in the implantation rate or pregnancy outcomes, such as biochemical pregnancy, clinical pregnancy, live birth and early abortion, between the two groups. In other words, the presence of CSD did not affect the pregnancy outcomes of embryo transfer in patients with previous CS. However, Wang et al. reported that CSDs decreased the pregnancy rate and implantation rate of embryos at the cleavage stage and reduced the live birth rate of blastocysts. However, their study has several limitations. First, the researchers did not separately analyze the pregnancy outcomes of fresh embryo transfer and frozen embryo transfer. Second, the sample size of the CSD group was small (58 and 40, respectively), which affects the credibility of the results. Finally, they did not adjust for various confounding factors on pregnancy outcomes (11). Another retrospective study conducted by Diao J et al. revealed that a uterine defect might have a detrimental effect on IVF/ICSI-ET outcomes (24). However, one limitation of the study was that they did not distinguish endometrial preparation, which might lead to more confounding factors and affect the outcomes (12).

Recently, some studies have reported that the presence of a CSD may decrease the pregnancy rate through the environment, physical barriers to embryo transfer and implantation, and psychogenic factors, which may reduce the likelihood of pregnancy (10, 23). The obvious changes at the scar site are leukopenia, decreased angiogenesis and delayed endometrial maturation, and the presence of scars affects endometrial receptivity (26, 27). A recent systematic review confirmed that the presence of a niche or CSD reduces live birth rates after ART, supporting these mechanisms (19). In addition, the size of the CSD was an important factor, with larger defects significantly lowering live birth rates even after single embryo transfer cycles (18). In this study, we found that diverticulum size did not affect the clinical pregnancy rate or live birth rate. However, the sample size of this subgroup was small, and we used RMT to represent the size of the diverticulum, which might reduce the accuracy and credibility of the research results. In the future, a larger sample size is needed to further study the influence of diverticulum size on pregnancy outcomes.

The advantage of this study is that in all patients who underwent IVF/ICSI-FET cycles, HRT was used to prepare the endometrium, and there was no statistically significant difference in endometrial thickness, which reduced the influence of confounding factors on pregnancy outcomes. Furthermore, the influence of CSDs on perinatal complications and neonatal outcomes was also analyzed. However, there are several limitations in this study. First, we did not select the first FET cycle in this study, and repeated transfers may affect pregnancy outcomes. Second, this was a retrospective study. Different reproductive doctors, embryologists, ultrasound doctors and ultrasound machines may affect the homogeneity of patients, thus affecting the results. Third, detailed data on the distribution of twin pregnancies were not consistently available in our study. As twin pregnancies may influence miscarriage rates and other obstetric outcomes, the absence of this information restricts our ability to evaluate their impact fully. Finally, all the data were from the same reproductive center, and the sample size of the study was small. This may also have a certain effect on the outcomes.

In conclusion, our study indicates that a prior CS affects pregnancy outcomes. This study suggests that for infertility patients with a previous CS, we should solve the fertility problem rather than the CSD problem. However, for patients with CSDs, we should strictly strengthen pregnancy monitoring and risk control, address pregnancy complications as soon as possible, and maintain pregnancy safety. In the future, we will conduct a prospective study, and the results of the study need to expand the multicenter collaboration of samples for further follow-up research.

## Data Availability

The original contributions presented in the study are included in the article/Supplementary Material, further inquiries can be directed to the corresponding author.
